# Comparison of physicochemical and volatile flavor properties of neon flying squid (*Ommastrephes bartramii*), jumbo squid (*Dosidicus gigas*), and Argentine shortfin squid (*Illex argentinus*) during chilled storage

**DOI:** 10.3389/fnut.2023.1195944

**Published:** 2023-05-15

**Authors:** Jiancong Huo, Hui Yao, Jiagen Li, Jinmei Wang, Soottawat Benjakul, Bin Zhang

**Affiliations:** ^1^Key Laboratory of Health Risk Factors for Seafood of Zhejiang Province, College of Food Science and Pharmacy, Zhejiang Ocean University, Zhoushan, China; ^2^Rongcheng Taixiang Food Co., Ltd., Rongcheng, China; ^3^Faculty of Agro-Industry, International Center of Excellence in Seafood Science and Innovation, Prince of Songkla University, Songkhla, Thailand

**Keywords:** squid mantle, myofibrillar proteins, stability, chilled storage, quality changes

## Abstract

The difference and similarities in the physicochemical and volatile flavor properties were determined in neon flying squid (OB), jumbo squid (DG), and Argentine squid (IA) mantles during 8 days of chilled storage. Physicochemical analysis indicated the chilled conditions induced rapid increases in pH value, total volatile basic nitrogen (TVBN), and carbonyl and malondialdehyde (MDA) content of the three squid species. In addition, myofibrillar protein (MP) content decreased and springiness in the OB, DG, and IA mantle samples declined with the extension of storage time. Importantly, OB mantles presented less chemical stability than the other two squid samples during 8 days of chilled storage. In addition, histological observations suggest DG mantle tissues presented more compact structures than those of the other two samples. Volatile flavor analysis showed propionaldehyde, 3-pentanone, trimethylamine, 3-furanmethanol, 2-methyl butyric acid, and 2-butanone were highly abundant in the squid mantles after storage, likely resulting from decomposition, oxidation, and degradation of proteins and lipids in the squid mantle, which varied with different squid species. The findings provide insight into the performance of three squid species during chilled storage.

## 1. Introduction

Squid is one of the most important aquatic products around the world, according to the international trade data shown in recent years (1, 2). The increasing demand for squid products has enhanced the economic importance of this industry in recent years, due to its distinctive organoleptic properties and high nutritional value. The flavor characteristics are primary factors determining the value of squid products. However, squid tends to produce unpleasant chemical substances (e.g., alcohols, ketones, and aldehydes) under supermarket display conditions (0–4°C), leading to the development of off-putting flavors and reduced economic value (3). The key aspect of squid processing and storage is the ability to maintain the quality of the products, and the investigation of the quality changes in squid during chilled storage provides a foundation for developing better storage methods for extending the shelf-life of squids.

The quality of squid begins to decline post-mortem because of specific microbiological and physicochemical reactions, which act through enzymatic hydrolysis and are activated by temperature and oxygen (4, 5). As a result, undesirable flavor substances, such as trimethylamine (TMA) and dimethylamine (DMA), begin to accumulate (6). In addition, structural changes in muscle proteins occur under chilled storage, along with the slow freezing of water molecules, resulting in a dull and opaque texture and a decrease in the edibility (7–9). The deterioration of squid quality is characterized by changes in sensory qualities associated with the skin and muscle color. These changes inspire the need for thorough investigations of squid products during chilled storage.

Currently, the valuable species of squid mainly include neon flying squid (*Ommastrephes bartramii*), jumbo squid (*Dosidicus gigas*), and Argentine shortfin squid (*Illex argentinus*). Regardless of species, squid are transported and landed under frozen condition. Most studies on squid products have mainly focused on the relationship between the storage conditions and properties of muscle proteins. In particular, the impacts of temperature fluctuations and the freezing method on commercial properties, such as color, shelf-life, and microbial growth, have been studied (10–14). Several studies focused on the physicochemical properties of jumbo squid muscle, especially the structural changes in tissue proteins (15, 16). However, the differences in the physicochemical and volatile flavor properties between different squid species during chilled storage have not been investigated.

In this study, three squid species (*O. bartramii*, *D. gigas*, and *I. argentinus*) were subjected to chilled conditions at 4°C for 8 days. The physicochemical and volatile flavor properties of three squids, including the changes in microstructures and textural properties, total volatile basic nitrogen (TVBN) content, pH, myofibrillar proteins (MP) and carbonyl content, and malondialdehyde (MDA) content, were investigated. The results of this study provide important information to improve the preservation of squid products.

## 2. Materials and methods

### 2.1. Chemical reagents

Thiobarbituric acid (TBA) and glutaraldehyde were purchased from Shanghai Source Poly Biological Technology Co., Ltd. (Shanghai, China). Bouins fixative solution (containing 75% saturated picric acid, 25% formalin solution, and 5% glacial acetic acid) was purchased from Beijing Solarbio Science and Technology Co., Ltd. (Beijing, China). Boric acid, methyl red, trichloroacetic acid (TCA), dinitrophenylhydrazine (DNPH), and other reagents (analytical grade) used in this study were obtained from Sinopharm Chemical Reagent Co., Ltd. (Shanghai, China).

### 2.2. Squid samples and treatments

Neon flying squid (*O. bartramii*; abbreviated as OB, average weight 500 ± 10 g), jumbo squid (*D. gigas*; abbreviated as DG, average weight 1,000 ± 25 g), and Argentine shortfin squid (*I. argentinus*; abbreviated as IA, average weight 400 ± 10 g) were purchased from Zhejiang Xingye Group Co., Ltd. (Zhoushan, China). All the squid samples were transported immediately to the laboratory in an ice box (0–2°C) within 20 min. Upon arrival, the squid individuals (TVBN content determined as 3.5–3.8 mg/100 g) were removed and washed with distilled water three times and then wiped with filter papers to remove water on the squid surface. The mantle was manually prepared by removing the tentacles, pen, viscera, skin, and head. After washing with distilled water, the obtained mantle samples were stored in a refrigerator at 4°C.

### 2.3. Springiness and histological analysis

Texture profile analysis (TPA) of the squid mantles was carried out using a texture analyzer (TA.XT PlusC, Stable Micro Systems, Godalming, United Kingdom) according to the method reported by Tabilo-Munizaga and Barbosa-Cánovas (17). The parameters were as follows: pre-test speed of 1.0 mm/s, test speed of 5.0 mm/s, post-test speed of 5.0 mm/s, trigger force of 5 g, and compression ratio of 50%. Springiness was calculated according to the force–time curves.

Scanning electron microscope (SEM) analysis was performed according to the method of Zhou et al. (18) with slight modifications. The squid mantle was cut into 3.0 × 3.0 mm sections from the same position in each sample. The muscle tissues were fixed in 2.5% (*v*/*v*) glutaraldehyde solution for 24 h. The histological changes (longitudinal direction) in muscle tissues were observed using a scanning electron microscope (SU3800, Hitachi Limited, Tokyo, Japan).

Hematoxylin–eosin (HE) and Masson staining analysis were carried out to evaluate the changes in the microstructure of muscle tissues according to the methods of Ando et al. (19) and Cavallaro et al. (20). Briefly, small muscle blocks (4.0 × 4.0 mm) were prepared from the longitudinal dorsal section of squid mantles. HE staining analysis was conducted as follows. The tissues were embedded in Davidson’s fixative mixture, consisting of distilled water, formalin, acetic acid, and alcohol. The fixed samples were then dehydrated with a series of ethanol solutions. After dehydration, the muscle tissues were embedded in paraffin and sectioned with a microtome into 5 μm transverse sections. Finally, the sections were stained in HE solution and histologically (longitudinal section) inspected under a light microscope (model CX23, Olympus Co., Ltd., Beijing, China). Masson staining analysis was performed as follows. The muscle blocks were fixed in a Bouins fixative solution and embedded in paraffin wax. Paraffin-embedded muscle blocks were cut into 2-μm-thick slides. The slides were stained according to Masson’s trichrome staining protocol and histologically (longitudinal section) inspected under a light microscope.

### 2.4. Total volatile basic nitrogen content and pH analysis

Total volatile basic nitrogen (TVBN) content was measured according to the method described by Xuan et al. (21). TVBN content was expressed as mg/100 g squid mantle. Briefly, 5 g of squid mantle tissues was homogenized with 20 ml of distilled water. The pH of the homogenate was measured using a pH meter at 25°C (FE28, Mettler Toledo, Zurich, Switzerland).

### 2.5. Myofibrillar protein and carbonyl content analysis

Myofibrillar protein (MP) extraction was prepared according to the method described by Zhang et al. (22) with minor modifications. Briefly, 10 g of squid mantle was weighed and homogenized with four volumes of buffer A (20 mmol/l Tris–HCl solution containing 0.1 mol/l KCl, pH 7.5). The mantle sample was centrifuged at 10000 × *g* for 20 min at 4°C. The precipitate was collected and extracted again. Subsequently, four volumes of buffer B (20 mmol/l Tris–HCl solution containing 0.6 mol/l KCl, pH 7.0) were added to the precipitate. The obtained mixture was homogenized for 2 min and then incubated at 4°C for 1 h. After centrifuged at 10,000 × *g* for 20 min, the supernatant was retained as the extracted MPs (mg/g).

The carbonyl content in the MPs was measured according to the procedures described by Uzun et al. (23). Briefly, the MP solution was precipitated with 10% (w/v) TCA solution. After centrifugation (10,000 × *g* for 20 min, at 4°C), the resulting sediment was redissolved in 2.0 mol/l HCl containing 2.0 g/l DNPH in the dark for 60 min (25°C). Then, 20% (*w*/*v*) TCA solution was added to the mixture, followed by the same centrifugation again. The obtained sediment was washed three times with ethanol/ethyl acetate (1:1, *v*/*v*) solution. Finally, the protein sediment was dissolved in 20 mmol/l sodium phosphate buffer (containing 6 mol/l guanidine) before the absorbance at 365 nm was measured. The carbonyl content was expressed as nmol of DNPH fixed/mg of MPs.

### 2.6. Malondialdehyde content analysis

The malondialdehyde (MDA) content in the squid mantle was measured using a MDA test kit (Nanjing Jiancheng Bio. Inst., NanJing, China). The squid mantle muscle was homogenized (1:9, *w*/*v*) in a normal saline solution, and the homogenates were centrifuged at 8,000 × *g* for 15 min at 4°C. The obtained supernatants were collected, and the MDA content was measured. The measurement was conducted according to the manufacturer’s instructions and expressed as nmol MDA per mg muscle.

### 2.7. Volatile compound analysis

Volatile compound analysis was carried out using a gas chromatograph (GC) coupled with a high-resolution ion mobility spectrometer (IMS; FlavourSpec^®^, Hanon Advanced Technology Group Co., Ltd., Jinan, China) equipped with a FS-SE-54-CB-1 chromatographic column (15 m in length and 0.53 mm in internal diameter) using N_2_ as a carrier gas. Ten grams of squid mantle was homogenized in a blender for 5 min. Then, 2 g of mashed muscle was transferred to a 20 ml headspace vial and incubated for 15 min at 60°C before collection. The testing procedures were as follows: injection volume of 500 μl; injection temperature of 85°C; and incubation rotation rate of 500 rpm. The gas chromatography conditions were as follows: column temperature of 60°C; analysis time of 20 min; initial carrier flow rate at 2 ml/min for 2 min, increased to 10 ml/min for 8 min, and finally increased to 100 ml/min for 10 min.

### 2.8. Data analysis

All determinations were measured in triplicate. The results obtained from the measurements were subjected to statistical analysis in SPSS package 13.0 for Windows (SPSS Inc., Chicago, IL, United States). Averages were compared via Duncan’s test, and a *p*-value of less than 0.05 indicated the means were significantly different.

## 3. Results and discussion

### 3.1. Springiness and histological analysis

**Figure 1:**
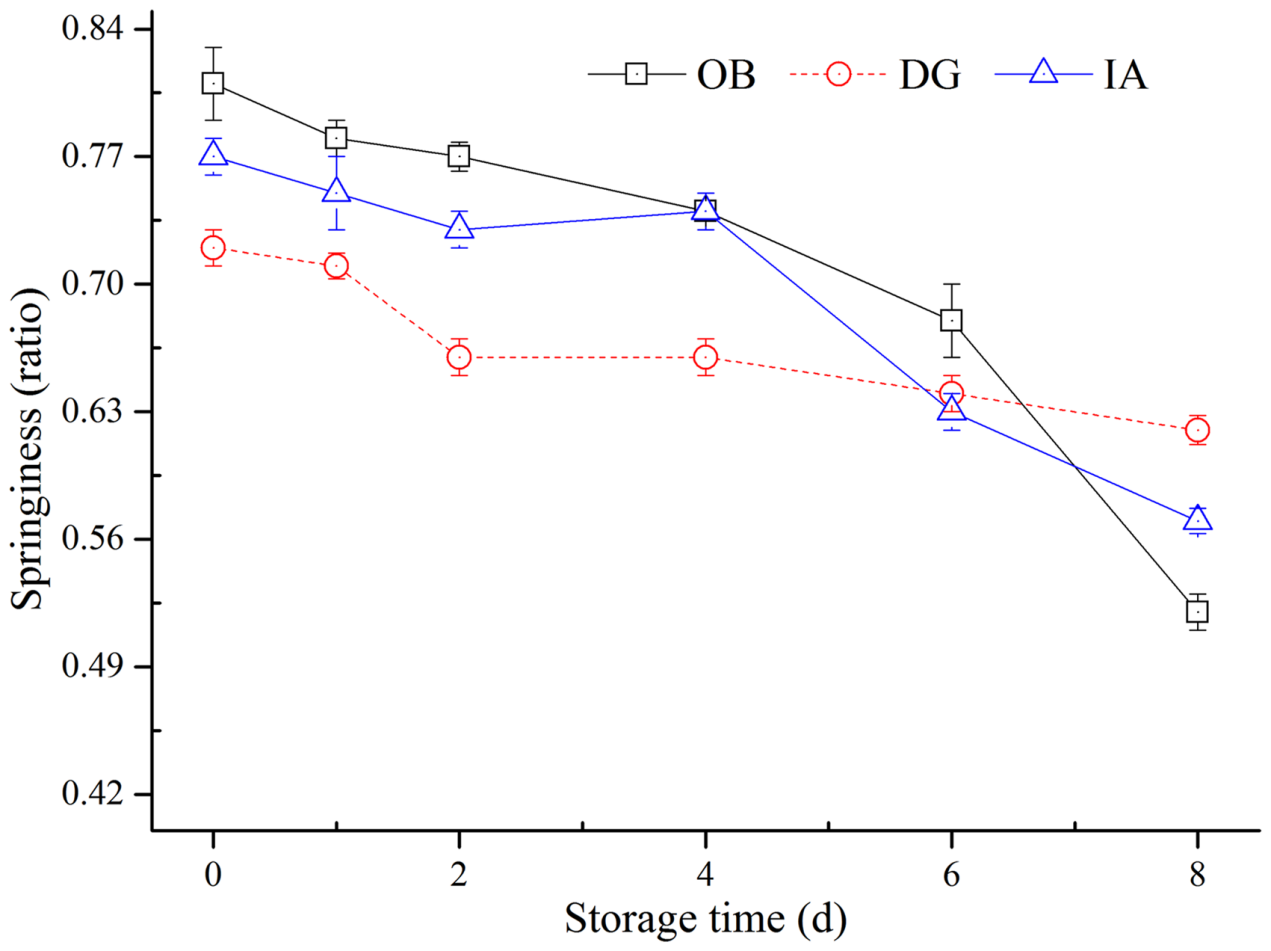
Changes in the springiness of OB, DG, and IA mantle tissues during 8 days of chilled storage.

The springiness of all three squid mantle muscle samples decreased during chilled storage according to Figure 1. After 8 days of chilled storage, the springiness values of OB, IA, and DG mantles were 0.524, 0.602, and 0.625, respectively. In particular, the decreasing rate of DG mantels was the slowest, compared with the other two samples. At the end of storage, 80% of initial springiness remained in DG mantels, but only approximately 60% of the original springiness remained in OB and IA mantels. The variations in muscle texture were closely related to the structure of muscle proteins. This was especially true for MPs, which play important roles in the stabilization of muscle texture. The improved stability of MPs could in turn brought significant maintenance effects on the histological properties (24–26). According to studies by Gokoglu et al. (27) and Hansen et al. (28), the textural characteristics, including springiness and chewiness, varied greatly according to the species, content of MPs, and the processing and storage conditions of squid samples. In the current study, there were differences in the content and structure of MPs, and this was mainly due to the different species of squid. In addition, the decrease in springiness was attributed to the denaturation and oxidation of muscle proteins, the growth of bacteria, and degradation via endogenous enzymes during chilled storage (29).

**Figure 2:**
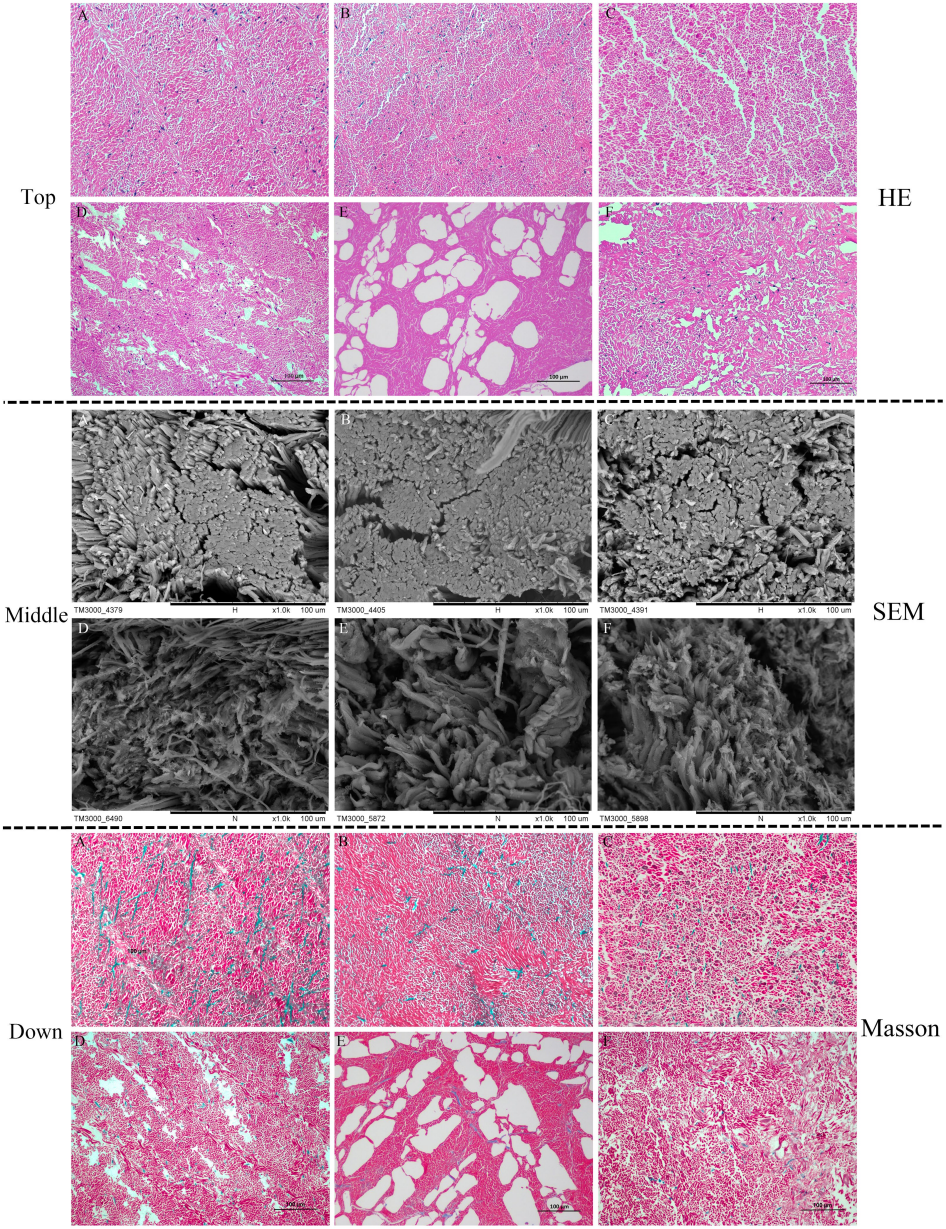
Hematoxylin–eosin (HE) staining (top), SEM (middle), and Masson staining (down) micrographs of squid mantle tissues (transversal section). **A–C**: fresh IA, OB, and DG mantle (0 days), respectively; **D–F**: IA, OB, and DG mantle stored at 4°C for 8 days, respectively.

HE staining detects changes in muscle fibers, especially MPs. The structure of muscle fibers is closely related to its elasticity, which is easily tended to be dissociated during processing and storage due to high or low temperature. Thus, the changes in muscle fibers contribute to the loss of water and a more serious deterioration in the muscle structure. As indicated in Figure 2 (top), the arrangement of myofibrils in fresh squid mantles was clearly ordered. The myofibrils were securely attached to each other, and no apparent space appeared between the muscle fibers. By contrast, after 8 days of storage, intercellular spaces appeared between the muscle fibers. Moreover, the dis-aggregation of muscle fibers was found in the OB mantles, which was more serious than in the other squid samples. Conversely, the arrangement of muscle fibers was still more compact on the 8th day in the DG mantles. These observations are in agreement with the SEM analysis.

The SEM images in Figure 2 (middle) show the muscle fibers of fresh squid were arranged in a compact manner, and there were no apparent differences between the three squid species. At the end of chilled storage, gaps and spaces appeared on the surface of muscle fibers in all three samples. However, the extent of deterioration differed among the different species. Specifically, the fiber bundles appeared relatively compact and intact in the DG mantles, which were much better than the IA and OB samples. These observations correlated with the higher springiness of DG mantles, compared with the other squid samples.

The Masson staining results in Figure 2 (bottom) show the collagen (blue staining) was uniformly distributed around myofibrils (red staining) in the three squid muscle tissues, and they were closely connected with each other. No apparent differences were observed between the squid mantle samples. After 8 days of chilled storage, the size of the collagen droplets was greatly reduced, the space between myofibrils was distinctly enlarged, and the myofibrils were disordered and irregular in the IA and OB samples. Notably, the collagen was maintained and fewer extracellular spaces and cracks were observed in the DG mantle, when compared with the IA and OB samples. Thus, the chilled conditions greatly affected the histological structure of muscle tissues. The decreased binding force between myofibrils and collagen and the degradation/disaggregation of muscle proteins negatively impacted the textural characteristics of squid mantle (30). The DG muscle tissues were tight and compact with higher collagen content, and this likely contributed to the higher springiness property of muscle tissues after 8 days of chilled storage.

### 3.2. Total volatile basic nitrogen content and pH analysis

**Figure 3:**
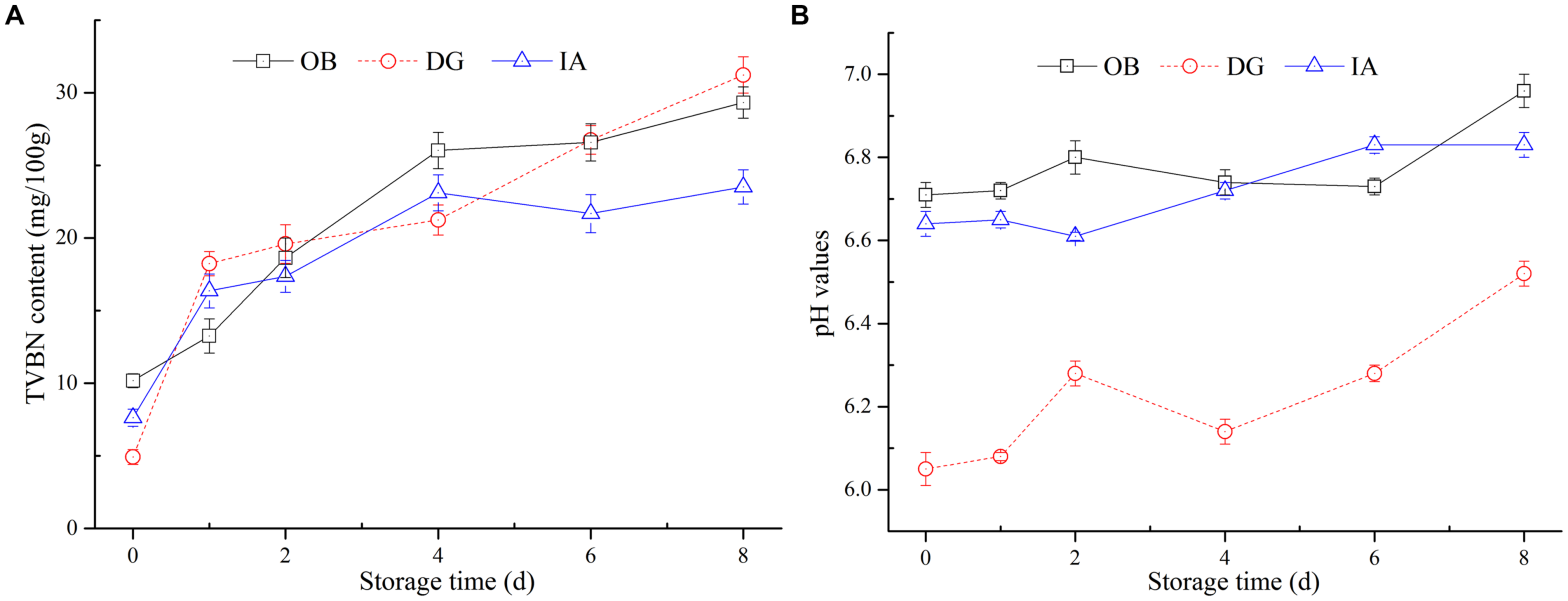
Changes in the total volatile basic nitrogen (TVBN) content **(A)** and pH value **(B)** of OB, DG, and IA mantle tissues during 8 days of chilled storage.

Total volatile basic nitrogen content is commonly used as an indicator of freshness and quality of muscle products, and it is associated with the activity of endogenous enzymes and bacteria. Figure 3A summarizes the effects of chilled storage on the TVBN values of three squid mantles during chilled storage. All squid samples exhibited significant increases in TVBN content. The TVBN content of IA and OB samples increased from 6.76 mg/100 g to 22.4 mg/100 g and 10.2 mg/100 g to 28.8 mg/100 g, respectively, over 8 days of storage, whereas the TVBN content of DG samples increased significantly from 5.0 mg/100 g to 32.4 mg/100 g. This value exceeded the limit of acceptability (30 mg/100 g) according to a report by Li et al. (31). Therefore, based on TVBN content, the IA mantle samples showed better stability than the DG and OB samples during 8 days of chilled storage.

The pH of muscle products was an indicator of acid–base balance and product quality. In Figure 3B, large fluctuations in pH values (6.06–6.56) appeared in the DG mantle muscle, suggesting the production of alkaline substances in the muscle tissues during storage. Interestingly, the pH values of OB and IA samples were relatively stable during storage, with increases from 6.70 and 6.62 to 6.88 and 6.82, respectively, and no significant differences (*p* > 0.05) were observed between the two samples. In general, the changes in pH were in accordance with the variations in the TVBN content of the three squid samples. The reason for this phenomenon was likely the formation of alkaline substances in the muscle tissues, which increased the pH values. Herein, the DG mantles showed the highest TVBN content and pH value at the end of storage, which might be due to the large size of DG squid samples. According to the findings by Vega-Gálvez et al. (32), the weight and size of squid are important factors that directly affected its ammonia and TVBN content. Similar findings were proposed by Fu et al. (33), who suggested the TVBN content in squid products was not only related to the processing and storage conditions but also dependent on the species.

### 3.3. Myofibrillar protein and carbonyl content analysis

**Figure 4:**
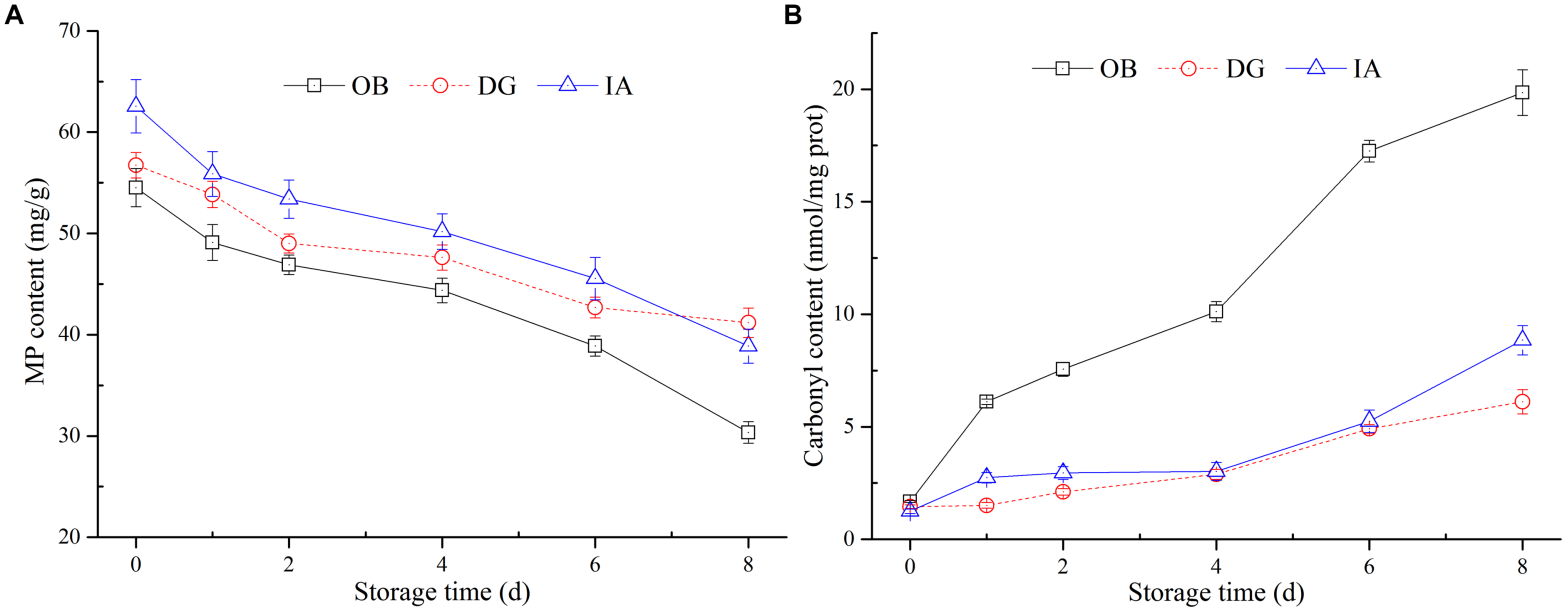
Changes in the myofibrillar protein (MP) **(A)** and carbonyl content **(B)** of OB, DG, and IA mantle tissues during 8 days of chilled storage.

Myofibrillar proteins are very important in maintaining the structure, integrity, and function of muscle tissues. To further examine the effects of chilled storage on squid muscle proteins, the MP content of three squid mantles was determined (Figure 4A). The results showed the MP content of all three squid samples decreased slowly with the extension of storage time. After 8 days of storage, the MP content of the three squid mantles was 30.4 (OB), 41.0 (IA), and 43.8 mg/g (DG). Compared with the initial MP content, the content decreased by 43.70, 34.92, and 23.15%, respectively. Among the three squids, the MP content of OB samples decreased at the fastest rate, followed by the IA and DG samples. During days 4–8 of storage, no significant differences in MP content were observed between IA and DG samples (*p* < 0.05), and the content was higher than that of the OB samples. This suggested the species of DG and IA were likely more stable during chilled conditions. In addition, the reactive sulfhydryls of MPs were oxidized to form disulfide bonds, resulting in the polymerization of myosin heavy chains, which might also lead to significant decreases in the MP content (34).

Carbonyl content is an important indicator of the extent of oxidative modification of muscle proteins. As oxidation continued, the spatial conformation of proteins was altered, the amino acid groups were modified, and the sulfhydryl groups buried in the MP interior were exposed, which induced the dissociation and degradation of MPs during storage (35, 36). In the current study (Figure 4B), the carbonyl content of OB mantles significantly increased to 20.4 nmol/mg after 8 days of storage and was significantly different from the other two samples (*p* < 0.05). This suggests a high degree of protein oxidation occurred in the OB mantle. In comparison, the carbonyl content of IA and DG samples was maintained at 7.46 and 6.22 nmol/mg at the end of storage, respectively, and no significant differences were observed between the two samples, except on the 8th day. These results are in agreement with the previous findings by Fuente-Betancourt et al. (37), who reported the MP content of jumbo squid tended to decrease during cold storage. According to the histological analysis, the DG mantle showed better stability during chilled storage, whereas the OB mantle tissues presented numerous small fragments and an irregular structure. These changes likely promoted the development of protein oxidation in the OB mantle tissues. Thus, the histological results are in agreement with the determination of MP and carbonyl contents in the IA, OB, and DG mantle samples.

### 3.4. Malondialdehyde content analysis

**Figure 5:**
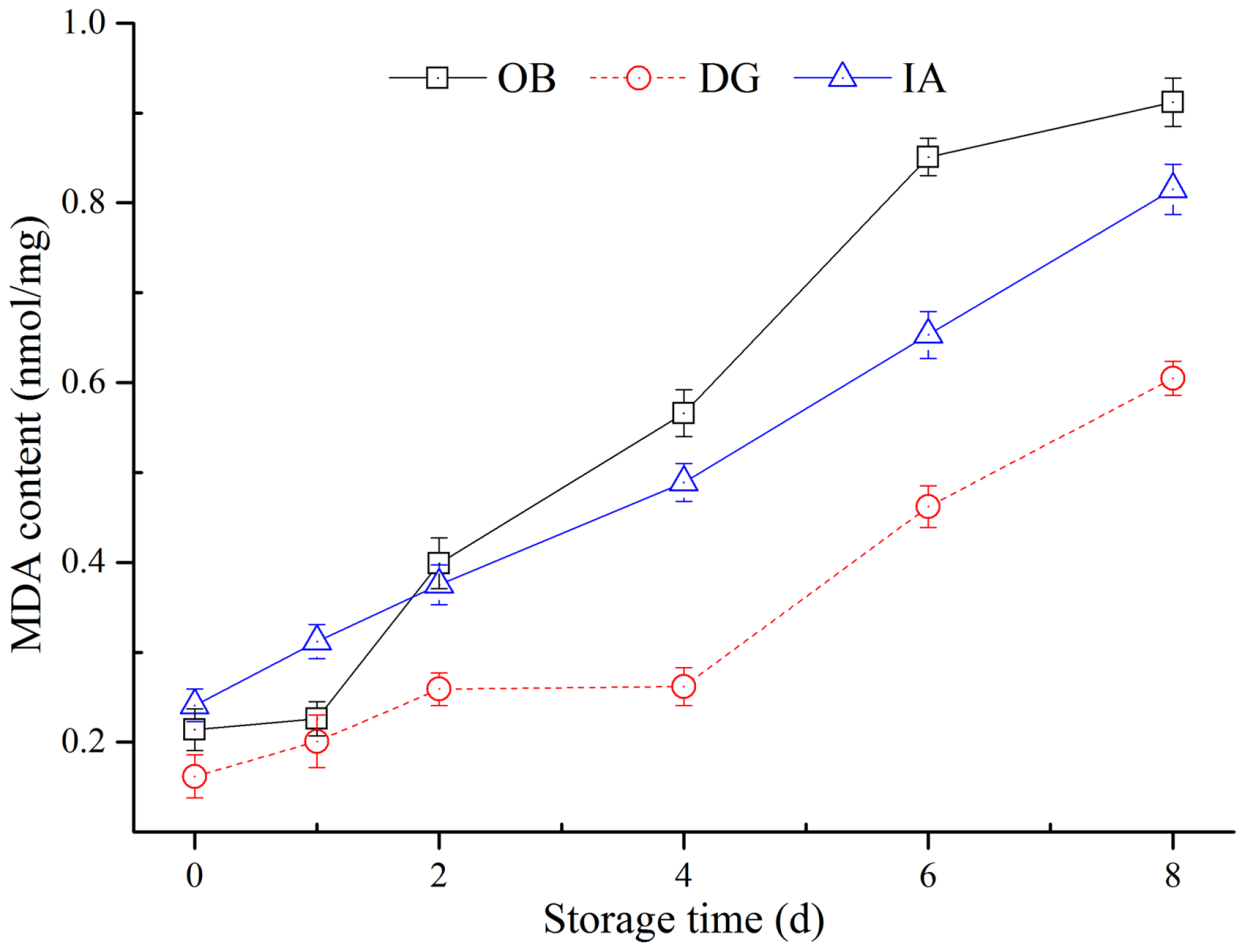
Changes in the malondialdehyde (MDA) content of OB, DG, and IA mantle tissues during 8 days of chilled storage.

Malondialdehyde content was determined to evaluate the degree of lipid oxidation in the three squids, and the results are presented in Figure 5. The MDA content of the OB, IA, and DG mantles increased from 0.22, 0.26, and 0.14 nmol/mg to 0.92, 0.84, and 0.56 nmol/mg during 8 days of storage, respectively. The MDA content of the DG samples was significantly lower (*p* < 0.05) than that of the OB and IA samples during 2–8 days of storage. The increase in the MDA content in all three squid samples was attributed to the oxidation of unsaturated fatty acids in the muscle tissues. These results are supported by a previous study by Dong et al. (38), who suggested the content of MDA is greatly affected by the presence of antioxidants (tea polyphenols), which inhibited the oxidation of unsaturated fatty acids in dried, seasoned squid (*D. gigas*) during chilled storage.

Lipid oxidation, as evaluated by MDA content, is closely correlated with rancid odors and off-flavors of squid products (21, 39). According to the MDA determinations, the DG mantle showed better lipid stability, compared with the IA and OB mantles, which was likely due to the low content of unsaturated fatty acid in the DG mantles. According to our previous results, the lipid content of the three squids differed, and the DG mantles had the highest lipid content but the lowest unsaturated fatty acid content (data not shown). These results most likely explained the significant differences in the MDA content among the three squids during chilled storage. Furthermore, it was well known that MDA is harmful to human health. Therefore, this study provides important findings regarding the refrigeration temperature and the packaging of squid products to reduce the content of MDA, especially for squid species with high lipid and unsaturated fatty acid contents.

### 3.5. Volatile compound analysis

**Figure 6:**
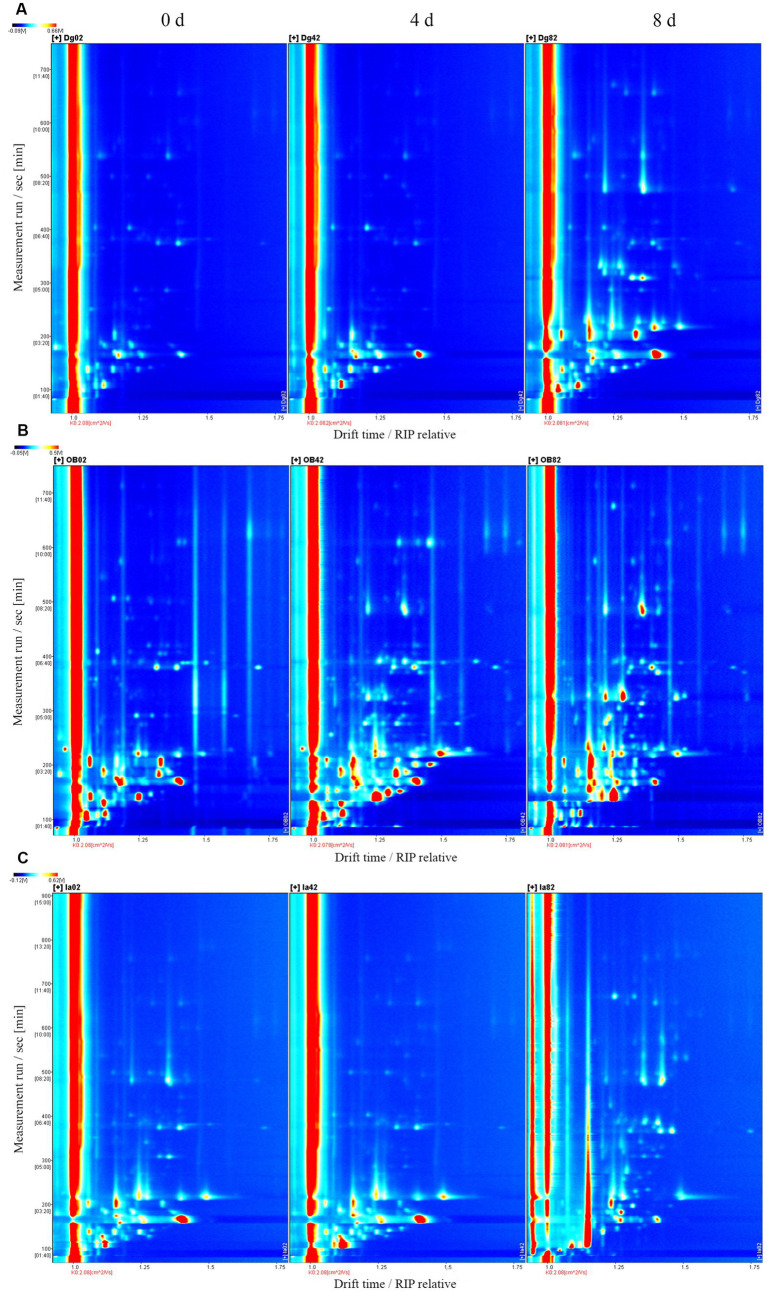
Topographical plots corresponding to GC-IMS signals detected in DG **(A)**, OB **(B)**, and IA **(C)** mantles on days 0, 4, and 8 of chilled storage, respectively.

The volatile organic compounds (VOCs) in the three squid samples were detected and identified during chilled storage by gas chromatography-ion mobility spectrometry. In the two-dimensional spectrum (Figure 6), the white color represents the low signal strength (intensity) of the VOCs, and the red color represents the high signal strength (intensity) of the VOCs in the squid samples. The darker color indicates greater intensity. A large number of VOCs were generated and detected in the squid mantles as storage time increased. This suggests the gradual deterioration in the volatile flavor characteristics and muscle quality in the squid mantles. For the OB samples, the composition and content of several VOCs were significantly different, compared with the DG and IA samples, likely resulting from the growth and reproduction of bacteria, oxidation of lipids, and hydrolysis of endogenous enzymes in the muscle tissues during chilled storage. These observations also confirmed the flavor quality of OB squid was less stable under chilled conditions.

**Figure 7:**
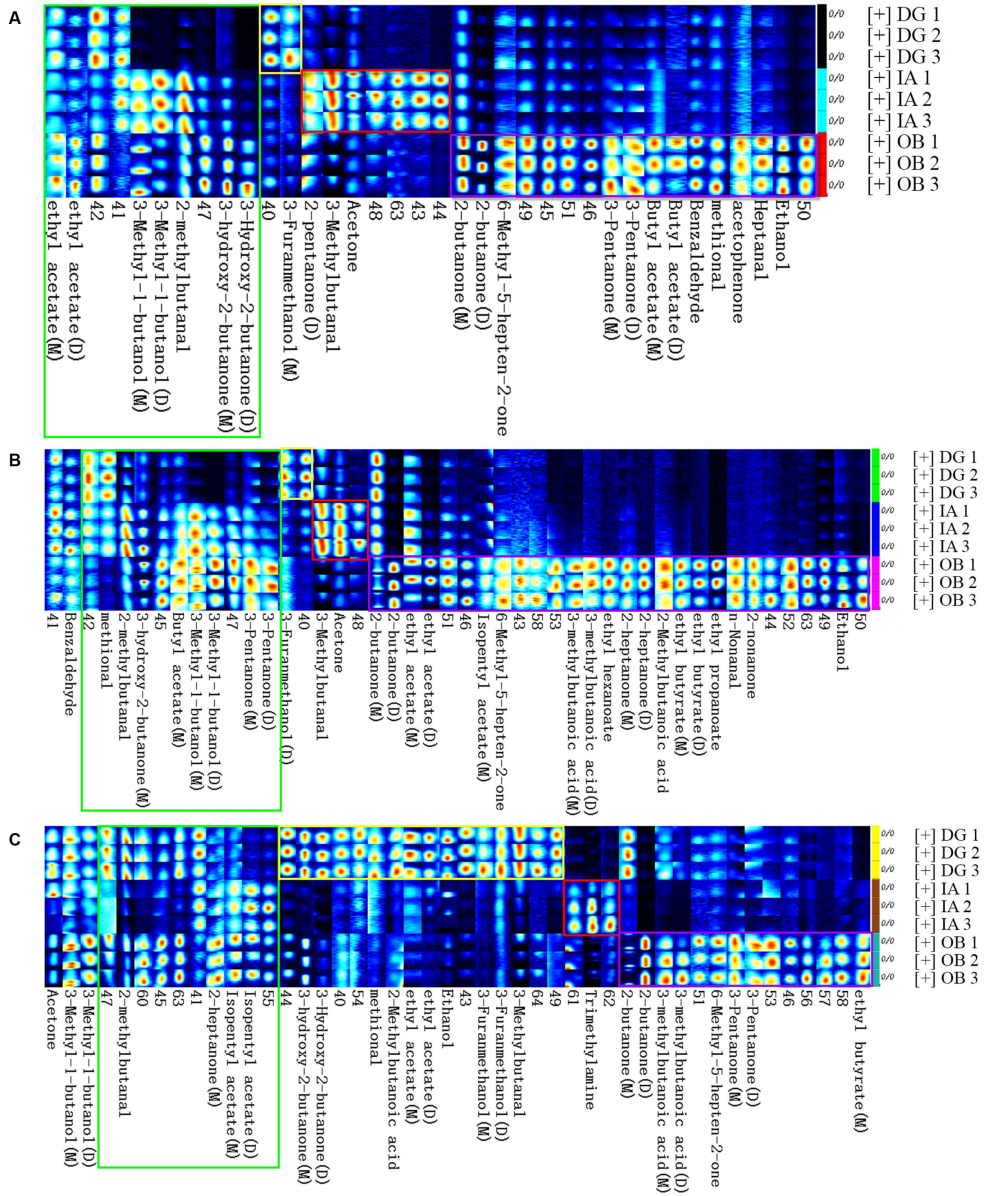
Fingerprint comparisons of volatile compounds detected in the DG, OB, and IA mantles on 0 **(A)**, 4 **(B)**, and 8 days **(C)** of chilled storage, respectively. Each row (spots) represents all the signals detected in a sample. Each column (spots) represents the same volatile compounds in different samples. Each group was analyzed in duplicate.

The fingerprinting plot (Figure 7) of these VOCs further depicts the differences between different squid samples. A total of 39 VOCs (except the unidentified VOCs, 25 compounds) were identified in the squid samples, including 4 aldehydes, 13 ketones, 7 alcohols, 10 esters, 4 acids, and 1 benzene. For the fresh squid samples (Figure 7A), high abundances of ethanol, heptanal, acetophenone, methional, benzaldehyde, butyl acetates, 3-pentanones, 6-methyl-5-hepten-2-one, and 2-butanones were detected in the OB mantles. Acetone, 3-methylbutanal, and 2-pentanone were found in higher abundances in the IA samples, and the DG samples showed a higher abundance of 3-furanmethanol. After 4 days of chilled storage (Figure 7B), significant variations in the VOCs could be found in the OB mantles, compared with the other squid samples, which indicated several chemical reactions occurred during the chilled storage. The IA mantles presented higher contents of acetone and isovaleraldehyde than the other two samples. In addition, 3-furanmethanol was found in high abundance in the DG mantles. Overall, the profiles, compositions, and content of VOCs in the chilled squid (4 days) were similar to those of the fresh squid samples. The fresh squid mantles presented comparatively good muscle quality, although the flavor characteristics decreased to a certain degree on day 4. After 8 days of storage (Figure 7C), a large number of VOCs, e.g., propionaldehyde, 3-pentanone, 6-methyl-5-hepten-2-one, isovaleraldehyde, trimethylamine, 3-furanmethanol, 2-methyl butyric acid, and 2-butanone, were detected in the three squid mantles. Some of these compounds exhibit unpleasant flavors and are derived from the decomposition, oxidation, and degradation of proteins and lipids in the muscle tissues (40).

In the current study, the differences in compositions and concentration of VOCs in the mantle muscle were closely connected with the species of squids when stored under the same conditions. Several fundamental volatile compounds were found in the three squid samples, but the compounds differed in each species. The VOCs were greatly affected by the storage period. For example, the aldehydes were derived from lipid oxidation (e.g., unsaturated fatty acids) and played important roles in the final volatile flavor of squid products. Notably, the content of aldehydes in the three squid varied with storage duration. In the DG mantles, aldehydes increased with storage time, and a decrease in the sensory qualities was also observed. However, the quality was considered acceptable, compared with the other two squids. In addition, the content of trimethylamine was higher in the IA mantles, indicating possible natural defects of the IA squid species. Importantly, the activity and growth of bacteria also contributed to the development of VOCs in the squid mantle muscle. Paarup et al. (41) reported cold-tolerant bacteria, such as *Photobacterium phosphoreum*, are involved in the formation of flavor compounds in squid (*Todaropsis eblanae*) during iced storage. More specifically, these microorganisms contributed to the formation of alkaline substances, which give rise to the formation of trimethylamine. The current findings indicate the importance of selecting the appropriate preservation conditions according to the species of the squid.

## 4. Conclusion

The physicochemical and volatile flavor properties of neon flying squid (*O. bartramii*), jumbo squid (*D. gigas*), and Argentine shortfin squid (*I. argentinus*) were investigated during 8 days of chilled storage. With the extension of storage time, the pH value, TVBN, carbonyl, and MDA contents of the three squid mantles significantly increased, and the springiness and MP content greatly decreased in all samples. The three squid species showed similar trends regarding these parameter. The DG mantles exhibited a rapid increase in pH, TVBN, and MDA content and slight decreases in MP content and springiness during storage. In addition, the carbonyl content of OB samples increased significantly during storage and was much higher than the other two squid samples. This indicates less stability of muscle proteins under chilled conditions. Moreover, the histological analysis suggests the DG mantle tissues presented tighter and more compact structures than those of the OB samples. Volatile organic compounds were also determined in the three squid samples, and alcohols, ketones, esters, and aldehydes were the most abundant. These compounds varied greatly between the squid species and with different storage durations. This study provides a thorough investigation of the similarities and differences in the physicochemical properties of three squid species during chilled storage and important insights for evaluating the performance of different squids during storage.

## Data availability statement

The original contributions presented in the study are included in the article/supplementary material, further inquiries can be directed to the corresponding author.

## Ethics statement

Ethical review and approval was not required for the animal study because the animal used in this study was regarded as a commercial marine resource and one of the most important aquatic products around the world. Therefore, ethical review is unnecessary for this study.

## Author contributions

JH was responsible for the design of experiment and the data collection. HY mainly contributed for the conduction of experiment. JW provided material. SB was helpful for the improvement of experiment. BZ was responsible for the revision of article. All authors contributed to the article and approved the submitted version.

## Funding

This study was funded by the Zhejiang Natural Science Foundation of China (No. LR21C200001) and the Zhejiang Leading Training Program of China (2020R52027).

## Conflict of interest

JW is employed by Rongcheng Taixiang Food Co., Ltd.

The remaining authors declare that the research was conducted in the absence of any commercial or financial relationships that could be construed as a potential conflict of interest.

## Publisher’s note

All claims expressed in this article are solely those of the authors and do not necessarily represent those of their affiliated organizations, or those of the publisher, the editors and the reviewers. Any product that may be evaluated in this article, or claim that may be made by its manufacturer, is not guaranteed or endorsed by the publisher.
